# Novel 4th-generation phytase improves broiler growth performance and reduces woody breast severity through modulation of muscle glucose uptake and metabolism

**DOI:** 10.3389/fphys.2024.1376628

**Published:** 2024-03-15

**Authors:** Carrie L. Walk, Garrett J. Mullenix, Craig W. Maynard, Elisabeth S. Greene, Clay Maynard, Nelson Ward, Sami Dridi

**Affiliations:** ^1^ DSM Nutritional Products, Kaiseraugst, Switzerland; ^2^ Department of Poultry Science, University of Arkansas, Fayetteville, AR, United States; ^3^ DSM Nutritional Products, Jerusalem, OH, United States

**Keywords:** broilers, phytase, growth, glucose uptake, GLUTs, AMPK, mTOR

## Abstract

The objective of the present study was to determine the effect of a novel (4th generation) phytase supplementation as well as its mode of action on growth, meat quality, and incidence of muscle myopathies. One-day old male broilers (*n* = 720) were weighed and randomly allocated to 30 floor pens (24 birds/pen) with 10 replicate pens per treatment. Three diets were fed from hatch to 56- days-old: a 3-phase corn-soy based diet as a positive control (PC); a negative control (NC) formulated to be isocaloric and isonitrogenous to the PC and with a reduction in Ca and available P, respectively; and the NC supplemented with 2,000 phytase units per kg of diet (NC + P). At the conclusion of the experiment, birds fed with NC + P diet were significantly heavier and had 2.1- and 4.2-points better feed conversion ratio (FCR) compared to birds offered NC and PC diets, respectively. Processing data showed that phytase supplementation increased live weight, hot carcass without giblets, wings, tender, and skin-on drum and thigh compared to both NC and PC diets. Macroscopic scoring showed that birds fed the NC + P diet had lower woody breast (WB) severity compared to those fed the PC and NC diets, however there was no effect on white striping (WS) incidence and meat quality parameters (pH, drip loss, meat color). To delineate its mode of action, iSTAT showed that blood glucose concentrations were significantly lower in birds fed NC + P diet compared to those offered PC and NC diets, suggesting a better glucose uptake. In support, molecular analyses demonstrated that the breast muscle expression (mRNA and protein) of glucose transporter 1 (GLUT1) and glucokinase (GK) was significantly upregulated in birds fed NC + P diet compared to those fed the NC and PC diets. The expression of mitochondrial ATP synthase F0 subunit 8 (MT-ATP8) was significantly upregulated in NC + P compared to other groups, indicating intracellular ATP abundance for anabolic pathways. This was confirmed by the reduced level of phosphorylated-AMP-activated protein kinase (AMPKα1/2) at Thr172 site, upregulation of glycogen synthase (GYS1) gene and activation of mechanistic target of rapamycin and ribosomal protein S6 kinase (mTOR-P70S6K) pathway. In conclusion, this is the first report showing that in-feed supplementation of the novel phytase improves growth performance and reduces WB severity in broilers potentially through enhancement of glucose uptake, glycolysis, and intracellular ATP production, which used for muscle glycogenesis and protein synthesis.

## Introduction

The US broiler industry is the largest producer of meat-type chickens, producing approximately 9.17 billion chickens in 2022 with a production value of $50,445,885,000 (USDA, 2023). Although it supports the livelihoods and food security of billions of people, the US broiler industry is facing several challenges from a steep projected increase in global demand for high quality animal protein by 73% by 2050; and the need to adapt to environmental constraints driven by climate changes and already scarce natural resources (water, energy, land). Additional challenges facing broiler production in the next decades will include, but are not limited to, water shortage, disease challenges and zoonoses, environmental footprint, and pollution concerns, while maintaining affordable, healthy, adequate, nutritious, and wholesome protein supply to feed the future of an ever-increasing world-population.

Poultry meat is highly regarded worldwide as one of the most efficient food sources with high quality nutrients, relatively inexpensive, and without religious taboos (Cavani et al., 2009; Marangoni et al., 2015). However, the newly emerging breast muscle disorders, woody breast (WB) (Bilgili et al., 2014) and white stripping (WS) (Bauermeister et al., 2009), have had an increasingly negative impact on worldwide chicken meat production and quality (Mudalal et al., 2015; Trocino et al., 2015). White stripping is characterized by visible white striations parallel to breast muscle fibers (Kuttappan et al., 2013), however WB is distinguished by a hard consistency to raw breast filets (Sihvo et al., 2014). Although probable different etiologies, both myopathies appeared in varying degree and more often together on the same breast filet. The lesions associated with both myopathies appear to be aseptic, superficially located, and include muscle fiber fragmentation, hyalinization, and swelling with replacement by fibrous connective tissue, as well as an influx of macrophages and other immune and fat cells (Soglia et al., 2016; Baldi et al., 2018). This constitutes a major animal health, welfare, and economic concern that costs the industry several hundred million dollars a year due to on-farm culling and mortality, down-grading, and condemnation at processing, as well as rejection from human consumption, and for which there is no effective solution because of its unknown etiology (Che et al., 2022). There is, therefore, a critical need to define its causation and identify mechanism-based strategies to prevent it.

Several high throughput omics studies postulated the involvement of diverse dysregulated cellular processes, including hypoxia, oxidative stress, and altered glucose utilization, in the progression of these muscle myopathies. Using functional mechanistic studies, Dridi’s group has shown that hypoxia induces WB through the unfolded protein response, ER stress, and modulation of satellite cell fate (Emami et al., 2021; Greene et al., 2023). Supplementation of high dose of phytase reduced the severity of WB via the improvement of local (muscle) and systemic oxygen homeostasis and modulation of muscle metabolome and fatty acid profile (Greene et al., 2019; Cauble et al., 2020; Greene et al., 2020). Furthermore, it has been reported that when fed at high dose, exogenous phytase induces a rapid and complete breakdown of diet-derived phytates, reduces the anti-nutritional effects, and thereby elicits a greater nutrient utilization, better growth (Shirley and Edwards, 2003; Rutherfurd et al., 2012), and free myo-inositol (Schmeisser et al., 2017; Gonzalez-Uarquin et al., 2020). Recently, a novel 4th generation phytase, HiPhorius™ (DSM Nutritional Products, Kaiseraugst, Switzerland), has been developed for optimal enzyme activity, thermo-resistance, stability to wide pH range in the gastrointestinal tract, and low phosphorus and greenhouse gas emissions (Imtiaz, 2022). It has been found to further improve broiler growth compared to classical phytase (Ronozyme HiPhos, DSM Nutritional products, Kaiseraugst, Switzerland) (Zhang et al., 2022). Yet, its mode of action is still not well defined. The aim of the present study was, therefore, to determine the effect and the mode of action of HiPhorius™ on growth performance, blood parameters, and muscle myopathy incidences in broiler chickens.

## Materials and methods

### Experimental design and diets

A total of 720 day-old straight run broiler chicks (Ross 708, Aviagen, Huntsville, AL) were obtained from a local commercial hatchery (Simmons Foods, Siloam Springs, AR) and transported to the University of Arkansas Broiler Research Farm. Upon arrival, chicks were randomly placed into 30 temperature-controlled pens at 24 birds per pen with a density of 0.096 m^2^/bird. Each pen was equipped with a hanging feeder, section of continuous nipple drinker line (5 nipples per pen), and built-up, top-dressed litter composed of clean pine shavings. All broilers were fed a 3-phase feeding program; d0-18 starter, d19-36 grower, and d37-56 finisher. Each pen was assigned to 1 of 3 isocaloric and isonitrogenous experimental treatments (10 pens/treatment), which consisted of a positive control with adequate nutrient supply (PC), negative control with a reduction of 0.08% Ca and 0.15% available P (NC), and a negative control supplemented with 2,000 phytase units/kg (FYT per kg of feed) of a novel fourth generation phytase [HiPhorius™, DSM Nutritional Products, Kaiseraugst, Switzerland, (NC + P)] (Table 1). Feed and water were provided *ad libitum* throughout the experiment. Initial temperatures were set at 32°C and gradually reduced to 20°C by d28 and remained there until the conclusion of the experiment. Lighting schedules were settled at 24L:0D for d0, 23L:1D from d1-6, and 18L:6D from d7-56. Light intensities were verified at bird level using a light meter (LT300, Extech Instruments, Waltham, MA). Birds were weighed and feed intake were measured by pen at d0, 18, 36, and 55 post hatch. Averaged body weight (BW), body weight gain (BWG), feed intake (FI), and mortality corrected feed conversion ratio (FCR) were subsequently calculated. These parameters and corresponding mortality were calculated for each phase and overall growth performance.

**TABLE 1:** Ingredients and nutrient composition of the experimental diets^a^.

Ingredient, %	Starter (0–18 days-of-age)		Grower (18–36 days-of-age)		Finisher (36–56 days-of-age)	
	PC	NC	PC	NC	PC	NC
Corn	52.39	53.50	57.04	58.14	61.17	62.28
Soybean meal	39.88	39.71	34.86	34.69	30.30	30.13
Poultry fat	3.45	3.06	4.15	3.77	4.94	4.56
Limestone	1.06	1.32	0.99	1.25	0.93	1.19
Dicalcium phosphate	1.85	1.03	1.62	0.81	1.40	0.58
Salt	0.22	0.22	0.22	0.22	0.22	0.22
Sodium bicarbonate	0.37	0.38	0.38	0.38	0.37	0.38
L-lysine HCl	0.14	0.14	0.13	0.14	0.11	0.12
L-threonine	0.05	0.05	0.03	0.03	0.01	0.01
DL-methionine	0.32	0.31	0.29	0.29	0.25	0.25
Vitamin premix^b^	0.10	0.10	0.10	0.10	0.10	0.10
Trace mineral premix^c^	0.10	0.10	0.10	0.10	0.10	0.10
Choline chloride, 60%	0.06	0.06	0.06	0.06	0.06	0.06
Coccidiostat	0.02	0.02	0.02	0.02	0.02	0.02
Calculated nutrient composition, %						
Dry matter	85.08	85.37	84.45	84.74	83.75	84.04
Crude protein	23.65	23.66	21.57	21.57	19.64	19.65
ME, kcal/kg	3,000.00	3,000.00	3,100.00	3,100.00	3,200.00	3,200.00
Total calcium	0.96	0.88	0.87	0.79	0.78	0.70
Total phosphorus (P)	0.71	0.56	0.65	0.50	0.60	0.45
Available P	0.48	0.33	0.44	0.29	0.39	0.24
Phytate P	0.27	0.27	0.26	0.26	0.25	0.25
Digestible lysine	1.28	1.28	1.15	1.15	1.02	1.02
Digestible threonine	0.86	0.86	0.77	0.77	0.68	0.68
Digestible methionine	0.63	0.63	0.58	0.58	0.52	0.52
Digestible SAA^d^	0.95	0.95	0.87	0.87	0.80	0.80
Sodium	0.20	0.20	0.20	0.20	0.20	0.20
Chloride	0.23	0.23	0.23	0.23	0.23	0.23
DEB^e^	252	252	232	232	214	214
Choline	1,700.00	1,700.00	1,700.00	1,700.00	1,700.00	1,700.00

^a^ PC, positive control; NC, negative control.

^b^ The vitamin premix contributed (per kg of diet): vitamin A, 30,864 IU; vitamin D3, 22,048 ICU; vitamin E, 220 IU; niacin, 154.32 mg; d-pantothenic acid, 39.68 mg; riboflavin, 26.44 mg; pyridoxine, 11.04 mg; thiamine, 6.16 mg; menadione, 6 mg; folic acid, 3.52 mg; biotin, 0.32 mg; vitamin B12, 0.04 mg.

^c^ The mineral premix contributed (per kg of diet): manganese, 100 mg; zinc, 100 mg; calcium, 69 mg; copper, 15 mg; iron, 15 mg; iodide, 1.2 mg; selenium, 0.25 mg.

^d^ SAA, sulfur amino acid.

^e^ DEB, dietary electrolyte balance.

### Blood parameters and tissue collection

On d55, 72 birds (8 pens/treatment; 3 birds/pen) were randomly selected, weighed, and sampled for venous blood analysis using an i-STAT Alinity system (SN:801128); software version JAMS 88.A.1/CLEW D44; Abaxis, Union City, CA, United States) with the i-STAT CG8^+^ cartridge test (ABBT-03P88-25) according to manufacturer’s recommendation. Heparinized whole blood (0.2 mL) was analyzed for ionized sodium (iNa), potassium (iK), chloride (iCl), calcium (iCa), total carbon dioxide (TCO_2_), glucose (Glu), bicarbonate (HCO^−^_3_), and pH. The i-STAT system has been validated in avian species (Martin et al., 2010; Schaal et al., 2016). Birds were euthanized by cervical dislocation immediately following blood draw and a cranial sample of left breast muscle (*Pectoralis major*) was removed, snap frozen in liquid nitrogen, and stored at −80°C until use.

### Processing

All birds underwent a feed withdrawal period of 10 h prior being transported to the University of Arkansas Pilot Processing Plant on d56. Five hundred and forty birds (18 birds/pen) were weighed to obtain live dock weights before being placed on shackles, electrically stunned (11 V, 11 mA for 11 s), exsanguinated, soft scalded (55°C for 2 min), de-feathered (Foodcraft Model 3; Baker international, MI, United States), and mechanically eviscerated. With-out giblets (WOG) and abdominal fat weights were then recorded, before being chilled for 3 h at 4°C. Chilled with-out giblets (CWOG) weights were recorded, before being deboned to acquire breast (*Pectoralis major*), tender (*Pectoralis minor*), wing, and leg quarter weights.

### Woody breast (WB) and white striping (WS) palpation and scoring

On d28 and 49, WB occurrence was estimated via live-bird palpation as previously described (Greene et al., 2019). After slaughter process at d56, breast filets were macroscopically scored and classified to WB and WS categories (Supplementary Figure S1). For WB and as described previously, a normal category (NORM) has a degree 0 with flexible breast throughout, moderate (MOD) has a degree 0.5–1.5 with mild hardening in the caudal area, and severe (SEV) has a degree with severe hardening and hemorrhagic lesions in the caudal region (Greene et al., 2019). For WS, a three-category scoring scheme, primarily accounting for the thickness and density of striations, has been used: normal (NORM) category is characterized by absence of white lines, moderate (MOD) with small (∼<1 mm), and severe (SEV) which is marked by large white striation (>1–2 mm) (Supplementary Figure S1) (Vignale et al., 2017).

### Meat quality

Breast fillets were collected after processing, placed on trays, and covered with plastic overlay before being stored at 4°C for 24 h for drip loss, pH, and colorimetric analysis. Drip loss (%) was calculated as the difference between hot debone breast weights and chilled breast weights and is expressed as a percentage of WOG. A Minolta colorimeter (CR-400; Konica Minolta Sensing Inc., Sakai Osaka, Japan; size 102(W) X 217 (H) X 63 (D) mm) using illuminant D65 and a 2-degree observer was used to determine the L* (lightness), a* (redness), and b* (yellowness) values from three readings on the ventral side of the left breast fillet. The same breast fillet was used to measure the average pH, from 3 readings, with a temperature-compensating pH meter (Testo 205; Testo Inc., West Chester, PA).

### RNA extraction, reverse transcription, and quantitative real-time PCR

Total RNAs were extracted from chicken left-breast muscle using Trizol reagent (Life Technologies, Carlsbad, CA) according to the manufacturer’s recommendations. After DNAse treatment and purification, the concentrations of total RNAs were measured for each breast muscle sample by Take 3 Micro-Volume Plate using Synergy HT multi-mode micro plate reader (BioTek, Winooski, VT), and RNA integrity and quality were assessed by both OD_260_/OD_280_ nm absorption ratio (>1.8) and by using 1% agarose gel electrophoresis. Reverse transcription and qPCR were performed as previously described (Dridi et al., 2012; Greene et al., 2023). In brief, RNA (1 µg) was reverse transcribed using qScript cDNA Synthesis Supermix (Quanta Biosciences, Gaithersburg, MD) in a 20 µL total reaction under the following conditions (42°C for 30 min followed by 85°C for 5 min). cDNAs were amplified by qPCR (Applied Biosystems 7500 Real Time System) with Power-Up Sybr green master mix (Life Technologies, Carlsbad, CA), 5 µL of 10X diluted cDNA, and 0.5 µ*M* of each forward and reverse specific primers as previously described (Dridi et al., 2012). Oligonucleotide primers specific for chicken mechanistic target of rapamycin (mTOR), ribosomal protein S6 kinase A1 (RPS6K), adenosine monophosphate-activated protein kinases (AMPKα1, AMPKα2, AMPKβ1, AMPKβ2, AMPKγ1, AMPKγ2, AMPKγ3), ribosomal RNA 18S and beta-actin have been previously reported (Nguyen et al., 2015; Vignale et al., 2015; Blankenship et al., 2016). Oligonucleotide primers specific for chicken glucose transporters (GLUT1, GLUT3, GLUT6, GLUT8, GLUT10, and GLUT12), glucokinase (GK), hexokinases (HK1, HK2, and HK3), glycogen synthases (GYS1 and GYS2), glycogen branching enzyme (GBE1), glycogen debranching enzyme (AGL), mitochondrial ATP synthase F0 subunit 8 (MT-ATP8), glycogen synthase kinase 3 beta (GSK-3β), eukaryotic translation initiation factor 2 alpha kinase 4 (EIF2AK4 or GCN2), eukaryotic translation initiation factor 2 subunit alpha (EiF2α), and eukaryotic translation initiation factor 4E binding protein 1 (EIF4EBP1) are summarized in Table 2. Relative expression of the target genes was determined using the 2^−ΔΔCT^ method (Schmittgen and Livak, 2008), with normalization to ribosomal 18S expression. PC broilers were used as a calibrator.

**TABLE 2:** Oligonucleotide QPCR primer**s**.

Gene^a^	Accession number^b^	Primer sequence (5′ → 3′)	Orientation	Product size (bp)
GLUT1	NM_205209	TCCTGATCAACCGCAATGAG	Forward	60
		TGCCCCGGAGCTTCTTG	Reverse	
GLUT3	NM_205511	TTGGGCGCTTCATTATTGG	Forward	68
		CTCACTGATGTACATGGGAACAAAG	Reverse	
GLUT6	XM_423637	CGCTTTGGACGTTGACATTG	Forward	62
		CTGGATGACTCGGGATGAGAA	Reverse	
GLUT8	NM_204375	GCTGCCTCAGCGTGACTTTT	Forward	58
		AGGGTCCGCCCTTTTGTT	Reverse	
GLUT10	XM_417383	AACGCAGAACAAAGATTCCTGAA	Forward	65
		GTCATTCCACGTGCCAGCTT	Reverse	
*GLUT12*	XM_419733	TTTGTGGACCTGTTTCGTTCAA	Forward	61
		GCGTGAGCCCTACCAGCAT	Reverse	
GK	AY256906	TGTTACATGGAGGAGATGCACAA	Forward	78
		GCCCCACTCCGTGTTCAC	Reverse	
HK1	NM_204101	TTATGTGGTGCCGGAATGG	Forward	61
		GCTCTAAGCCTCTGTTCTCCCTAA	Reverse	
HK2	NM_204212	AGAGCCCTCTGATGCCACAT	Forward	57
		GGAGGTGTCCGGAGAAAGG	Reverse	
HK3	XM_001231328	CACCGGAGAAACCTTGTGAGA	Forward	72
		CTGACTCGGCCATGAAGCA	Reverse	
GYS1	AB090806	GCCTCAACGTCCGCAAGTT	Forward	55
		TGGGCGTGCAGGTTCTG	Reverse	
GYS2	NM_001406729	CATTGACAAGGAAGCAGGAGAGA	Forward	67
		GTGTACAGATGCCCGTTCCA	Reverse	
*GBE1*	XM_015298401	AAGAAAATGGAGTATGGGAAATGG	Forward	71
		TGAGGCACAGGAGAAAAACCA	Reverse	
AGL	XM-040677682	TTCTAGCGTTTGGTGGGACTCT	Forward	58
		CCCTGGCCAAGCAGGTT	Reverse	
mtATP8	HM142824	CACACTTGCCGGAACGTACA	Forward	57
		GCCGTTTGCGTGGAGATT	Reverse	
GSK3α	XM_416557	AAGGCACATCCATGGACTAAGG	Forward	59
		GACCCGTACTCCTGAGGTGAAA	Reverse	
GCN2	XM_040671975	TGCGTCCTCAGGGATTGACT	Forward	66
		GGCACTTGACCCACAGATCA	Reverse	
EIF2S1	NM_001006477	GGCCGTCGCCAGAATG	Forward	59
		CCTCCGGAAACTTATGCTGGTA	Reverse	
EIF4EBP1	XM_424384	CTCTCCGTGTGGGTGTGAATAC	Forward	56
		CCCCACAGCCCATCATCA	Reverse	

^a^ AGL, glycogen debranching enzyme; EIF4EBP1, eukaryotic translation initiation factor 4E binding protein 1; EIF2S1, eukaryotic translation initiation factor 2 subunit alpha 1; GBE1, glycogen branching enzyme 1; GCN2, General Control Nonderepressible 2 or Eukaryotic Translation Initiation Factor 2 Alpha Kinase 4; GK, glucokinase; GLUT, glucose transporter; GSK3α, glycogen synthase kinase 3 alpha; GYS, glycogen synthase; HK, hexokinase; mtATP8, mitochondrial ATP, synthase F0 subunit 8.

^b^ Accession number refer to Genbank (NCBI).

### Western blot analysis

The immunoblot analyses were performed as previously described (Dridi et al., 2012; Emami et al., 2021; Greene et al., 2023). In brief, muscle tissues were homogenized in lysis buffer using bullet blender storm (NextAdvance, Averill Park, NY). Following concentration determination by a Bradford assay kit (Bio-Rad, Hercules, CA) using a Synergy HT multimode microplate reader (Biotek Agilent, Winooski, VT), total proteins (70–100 µg) were run in 4%–12% gradient Bis-Tris gels (Life Technologies, Carlsbad, CA) and then transferred to polyvinylidene difluoride (PVDF) membranes. After transfer, PVDF membranes were blocked using a Tris-buffered saline (TBS) with 5% nonfat milk and Tween 20 at room temperature for 1 h. The membranes were washed with TBS and Tween 20 and then incubated with primary antibodies at a dilution of either 1:500 or 1:1000 overnight at 4°C. Primary antibodies used were rabbit anti-glucose transporter 1 (GLUT1 #A6982, ABClonal, Woburn, MA), rabbit anti-GLUT12 (#LS-C110860, LSBio, Lynwood, WA), rabbit anti-glucokinase (GK, #A15059, ABClonal, Woburn, MA), rabbit anti-hexokinase 1(HK1, #A1054, ABClonal, Woburn, MA), rabbit anti-HK2 (#A20829, ABClonal, Woburn, MA), rabbit anti-phospho-AMP-activated protein kinase (AMPKα1/α2)^Thr172^ (#2531, Cell Signaling, Technology, Danvers, MA), rabbit anti-AMPKα1/α2 (#2603, Cell Signaling, Technology, Danvers, MA), rabbit anti-phospho-mechanistic target of rapamycin (mTOR)^Ser2448^ (#2971, Cell Signaling, Technology, Danvers, MA), rabbit anti-mTOR (#2972, Cell Signaling, Technology, Danvers, MA), goat anti-phospho-P70S6 kinase (P70S6K)^Thr389^ (#SC-11759, Santa Cruz Biotechnology, Dallas, TX), rabbit anti-P70S6K (#SC-230, Santa Cruz Biotechnology, Dallas, TX), mouse anti-phospho-glycogen synthase kinase 3 beta (GSK-3β)^Ser9^ (#SC-373800, Santa Cruz Biotechnology, Dallas, TX), and rabbit anti-Glyceraldehyde 3-phosphate dehydrogenase (GAPDH, #NB300-327, Novus Biologicals, Centennial, CO) as a housekeeping protein. Pre-stained molecular weight marker (Precision Plus Protein Dual Color) was used as a standard (BioRad, Hercules, CA) and as indicator for transfer efficiency. The secondary anti-mouse (#SC-358914, Santa Cruz Biotechnology, Dallas, TX)-, anti-goat (#SC-2354, Santa Cruz Biotechnology, Dallas, TX)-, and anti-rabbit (#7074S, Cell Signaling, Technology, Danvers, MA)-IgG-HRP-linked antibodies were used at 1:5,000 dilution for 1 h at room temperature. The signal was visualized by enhanced chemiluminescence (ECL plus) (GE Healthcare Bio-Sciences, Buckinghamshire, United Kingdom) and captured by FluorChem M MultiFluor System (Proteinsimple, Santa Clara, CA). Image Acquisition and Analysis were performed by AlphaView software (Version 3.4.0, 1993–2011, Proteinsimple, Santa Clara, CA).

### Statistical analyses

Data were analyzed by One-way ANOVA and Tukey’s HSD multiple comparison test using the Mixed Model platform of JMP Pro v. 17.0 (SAS Institute, Cary, NC, United States) for the growth, blood parameters, and meat quality and processing data, and Graph Pad Prism version 9.00 for Windows (Graph Pad Software, La Jolla California, United States) for muscle gene and protein expression profiles. For the growth data, pen served as the experimental unit and pen averages were calculated and analyzed for each quality characteristic. For the gene and protein expression, however, the bird was used as the experimental unit. For the muscle myopathy incidence, bird was the experimental unit and score was considered an ordinal variable. The model included diet. When diet was significant, score means between diets were separated using Pearson Chi-square. Statistical significance was set at *p* ≤ 0.05.

## Results

### Effect of phytase supplementation on growth performance

Phytase recoveries in the NC + P diet were 1,829; 1,595; and 1,771 FYT/kg in the starter, grower, and finisher phases, respectively. Broiler performance was approximately 83% of Ross 708 breed guidelines (Aviagen, Huntsville, AL) at d55. The overall mortality for the entire period (0–55 days) was high at 17.9% ± 7.5% and not influenced by treatment (*p =* 0.9798); the majority occurred from d18-55. This elevated mortality was a result of intense heat waves that happed on day 31 and 43 of the experiment (data not shown).

At the conclusion of the experiment (d55), birds fed with NC + P diet were significantly heavier and gained more (∼112 g/bird, *p* < 0.05) than the birds offered the PC and NCdiets (Table 3). There was no difference in feed intake between all studied groups, which resulted in 2.1- and 4.2-points better FCR for the birds fed NC + P diet compared to those fed NC and PC diets, respectively (Table 3). When categorized by rearing phase, birds fed NC + P diet had better FCR than their counterparts fed NC and PC diets at all periods (Tables 3), but the difference was statistically discernible only at starter period (Table 3). Birds fed the NC diet ate less feed (*p* < 0.05), gained less, and were significantly lighter compared to those offered the PC and NC + P diets at the starter phase, then they compensated and catch-up similar growth as the birds fed the PC diet during the grower and finisher periods (Tables 3).

**TABLE 3:** Growth performances during the entire experiment and by rearing phase^a^.

Treatment^b^	BW, g	BWG, g	FI, g	FCR, g:g	Mortality, %
Period (0–55 days)					
PC	3,475^b^	3,437^b^	5,853	1.705	17.92
NC	3,476^b^	3,438^b^	5,787	1.684	18.20
NC + P	3,588^a^	3,550^a^	5,893	1.663	17.58
SEM	0.036	0.036	0.049	0.014	2.20
*p*-value	0.039	0.044	0.322	0.138	0.979
Starter (0–18 days)					
PC	664^a^	626^a^	770^a^	1.234^a^	0.42
NC	633^b^	595^b^	726^b^	1.225^a^	0.00
NC + P	682^a^	644^a^	759^a^	1.190^b^	0.42
SEM	0.007	0.007	0.008	0.007	0.352
*p*-value	0.0003	0.0003	0.002	0.0005	0.612
Grower (18–36 days)					
PC	2,178^ab^	1,514	2,238	1.478	8.75
NC	2,126^b^	1,493	2,191	1.467	8.29
NC + P	2,207^a^	1,526	2,247	1.474	10.01
SEM	0.017	0.012	0.017	0.007	2.04
*p*-value	0.003	0.145	0.609	0.531	0.827
Finisher (36–55 days)					
PC	3,475^b^	1,296	2,845	2.210	8.76
NC	3,476^b^	1,350	2,869	2.144	9.91
NC + P	3,588^a^	1,381	2,887	2.117	7.13
SEM	0.036	0.035	0.037	0.047	1.94
*p*-value	0.039	0.243	0.734	0.377	0.601

^a^ BW, period averaged individual body weight; BWG, averaged individual body weight gain; FI, averaged individual feed intake; FCR, mortality corrected feed conversion ratio.

^b^ PC, positive control; NC, negative control; NC + P, negative control supplemented with phytase at 2,000 FYT/kg diet. Data are means of 24 birds per pen and 10 replicate pens per treatment. Different superscript letters within the same column indicate a significant difference at *p* < 0.05.

### Effect of phytase supplementation on carcass parameters and meat quality

As depicted in Table 4, phytase supplementation significantly increased live weight at processing, wings, and skin-on drum and thigh (LQ) compared to birds fed both NC and PC diets. Chickens fed NC + P diet exhibited a significantly higher tender weight compared to those offered the PC diet (Table 4). Phytase supplementation tended to increase WOG and CWOG weights compared to PC and NC diets (*p* = 0.05, Table 4). Although it was not statistically significant, birds fed NC + P diet exhibited higher breast weight (17 and 13 g) compared to those fed the PC and NC diets (Table 4). There was no significant difference in abdominal fat content between the studied groups (Table 4).

**TABLE 4:** Processing weights of 56 days-old Ross 708 broilers^a^.

Treatment^b^	BW, g	WOG, g	CWOG, g	Fat, g	Breast, g	Tender, g	Wings, g	LQ, g
PC	3,389^b^	2,687	2,774	45	786	163^b^	270^b^	838^b^
NC	3,372^b^	2,679	2,758	42	790	167^ab^	270^b^	832^b^
NC + P	3,514^a^	2,783	2,867	45	803	172^a^	281^a^	876^a^
SEM	38	31	32	1.3	12	2.2	2.9	10
*p*-value	0.035	0.050	0.052	0.265	0.561	0.043	0.023	0.012

^a^ BW, 56 days dock body weight.

^b^ WOG, hot carcass weight with-out giblets; CWOG, chilled carcass weight with-out giblets; Fat, hot fat pad; Breast, *pectoralis major*; Tender, *pectoralis minor*; Wings = skin-on wings; LQ, skin-on drum and thigh.

^2^PC, positive control; NC, negative control; NC + P, negative control supplemented with phytase at 2,000 FYT/kg diet. Data are means of 18 birds per pen and 10 replicate pens per treatment. Different superscript letters within the same column indicate a significant difference at *p* < 0.05.

**FIGURE 1:**
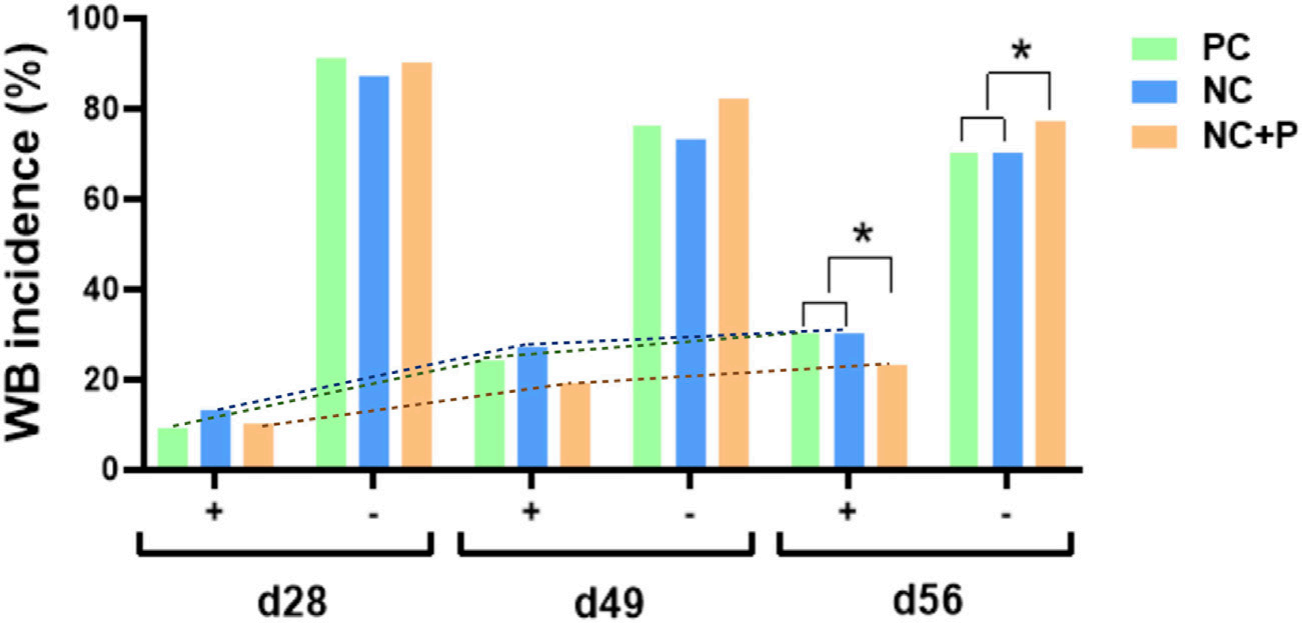
Effect of phytase supplementation on WB incidence in broilers at different ages. WB occurrence and incidence were assessed by live-bird palpation at the age of 28 and 49 days, and macroscopically scored at the processing (56 days). Data are presented as WB presence (+) or absence (−). WB incidence increased with age in all studied groups, but the amplitude was significantly lower in the NC + P birds particularly at d49 and d56. NC, negative control; NC + P, negative control supplemented with 2,000 phytase units/kg (FYT per kg of feed); PC, positive control.

There was no effect of treatment on the incidence of WS scores and incidence (Table 5). The combination of live-bird palpation and macroscopic scoring showed that WB incidence increased with age in all studied groups, but the amplitude was significantly lower in birds fed the NC + P diet particularly at d49 and d56 (Figure 1) compared to the other groups. Table 6 showed that phytase sypplementation reduced the WB severity (SEV, *p* = 0.0531) compared to PC and NC diets. Breast meat quality parameters (pH, drip loss, lightness, redness, and yellowness) were not influenced by the dietary treatments (Table 7).

**TABLE 5:** White stripping incidence in 56 days-old Ross 708 broilers^a^.

Treatment^b^	Average	Incidences of white striping, %		
		NORM	MOD	SEV
PC	0.393	68	24	8
NC	0.414	67	24	9
NC + P	0.405	68	23	9
SEM	0.050	3	2	2
*p*-value	0.978	0.984	0.980	0.871

^a^ A three-category scoring scheme, primarily accounting for the thickness and density of striations, has been used: degree 0 is a normal (NORM) category, which is characterized by absence of while lines, degree is a moderate (MOD) category with small (∼<1 mm) white striation, and degree 2 is severe (SEV) category, which is marked by large white striation (>1–2 mm).

^b^ PC, positive control; NC, negative control; NC + P, negative control supplemented with phytase at 2,000 FYT/kg diet. Data were obtained from 180 birds/group.

**TABLE 6:** Woody breast incidence in 56 days-old Ross 708 broilers^a^.

Treatment^b^	Average	Incidences of woody breast, %		
		NORM	MOD	SEV
PC	0.376	70	23	7
NC	0.357	70	24	6
NC + P	0.258	77	21	2
SEM	0.0469	3.7035	3.0676	1.4183
*p*-value	0.208	0.352	0.737	0.053

^a^ Scored on a numeric scale from 0–3: 0 = no woody breast; 1 = mild woody breast; 2 = severe woody breast. Data are means of 18 birds per pen and 10 replicate pens per treatment.

^b^ Means within the same column with different superscripts are significantly different at *p <* 0.05 and tendencies are presented at *p <* 0.10.

**TABLE 7:** Breast meat quality of 56 days-old Ross 708 broilers^a^.

Treatment^c^	pH	Drip loss, %	Breast fillet colour^b^		
			L*	a*	b*
PC	5.92	0.16	57.55	2.25	7.07
NC	5.93	−0.19	56.20	2.80	6.77
NC + P	5.93	0.36	56.93	2.48	7.24
SEM	0.03	0.17	0.72	0.30	0.37
*p*-value	0.975	0.099	0.437	0.458	0.673

^a^ Parameters were measured 24-h post-harvest.

^b^ L*–lightness; a*–redness; b*–yellowness.

^c^ PC, positive control; NC, negative control; NC + P, negative control supplemented with phytase at 2,000 FYT/kg diet. Data are means of 18 birds per pen and 10 replicate pens per treatment.

### Effect of phytase supplementation on blood parameters

There was no effect of dietary treatment on the concentration of Na, Ca, total CO_2_, or bicarbonate in the whole blood. However, birds fed the NC + P diet had a greater concentration of K (*p* = 0.0083) in the blood compared with birds fed the NC, and intermediate in birds fed the PC. Blood glucose (*p* = 0.037) and pH (*p* = 0.018) were lower in birds fed the NC + P diet compared with birds fed the PC and NC diets, respectively (Table 8).

**TABLE 8:** Blood parameters of 56-old Ross 708 broilers^a^.

Treatment^b^	BW	Na	K	Cl	Ca	TCO_2_	Glucose	HCO_3_	pH
	kg	mmol/L					mg/dL	mmol/L	
PC	3.65	150	5.92^ab^	114	1.43	24.3	254^a^	23.2	7.39^ab^
NC	3.79	149	5.70^b^	112	1.41	25.3	247^ab^	24.1	7.42^a^
NC + P	3.73	151	6.21^a^	115	1.44	24.6	240^b^	23.5	7.36^b^
SEM	0.10	0.81	0.11	0.83	0.001	0.52	3.7	0.52	0.01
*p*-value	0.646	0.209	0.008	0.093	0.320	0.346	0.037	0.487	0.017

^a^ Data obtained from i-Stat devices and report ionized minerals from 24 birds per treatment (3 birds per pen and 8 replicates per treatment).

^b^ PC, positive control; NC, negative control; NC + P, negative control supplemented with phytase at 2,000 FYT/kg diet. Different superscript letters within the same column indicate a significant difference at *p* < 0.05.

### Effect of phytase supplementation on muscle gene and protein expression profile

**FIGURE 2:**
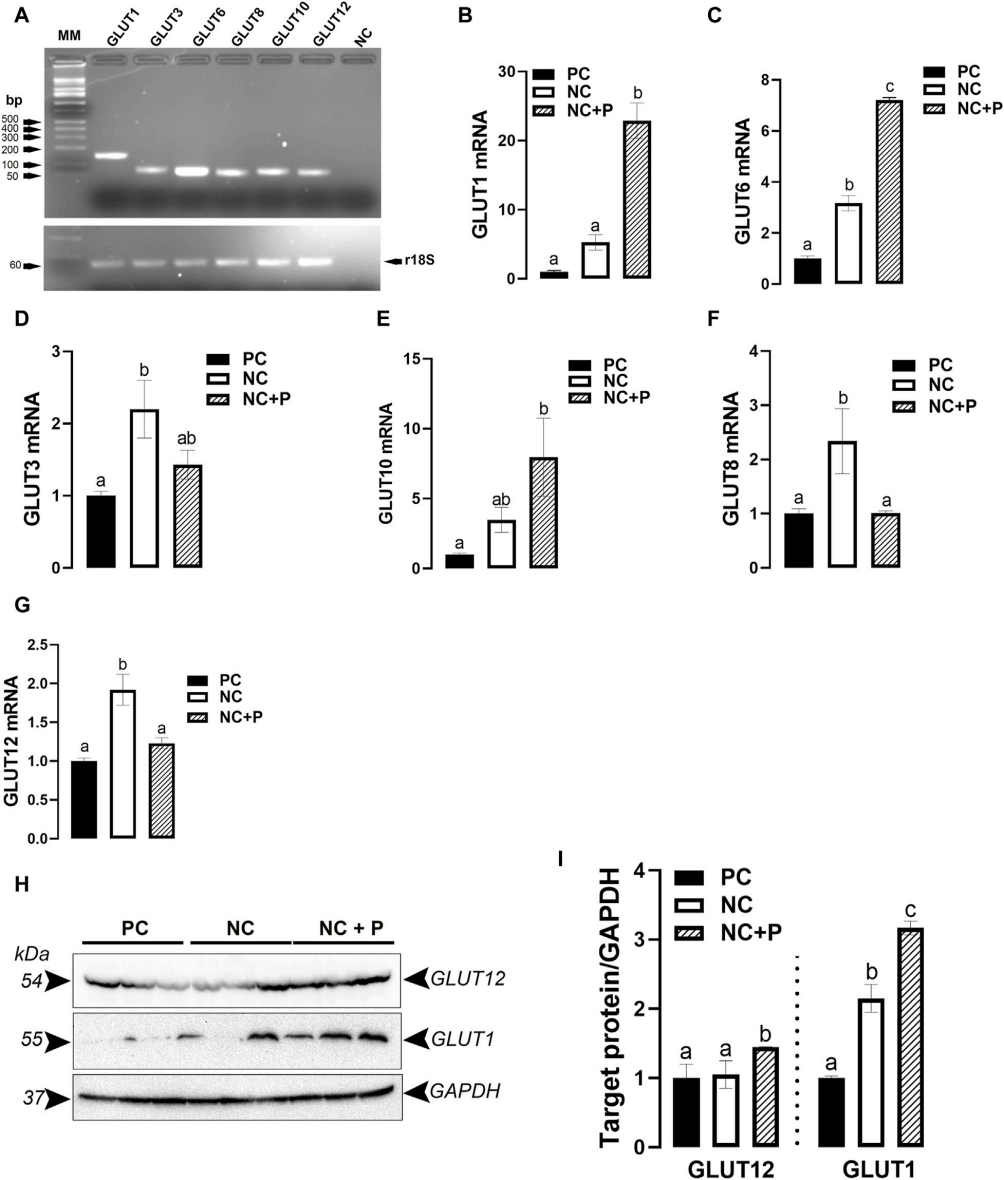
Effect of phytase supplementation on the expression profile of breast muscle glucose transporters (GLUTs). Several GLUT genes are expressed in the breast muscle **(A)**. mRNA abundances of GLUT1 **(B)**, GLUT6 **(C)**, GLUT3 **(D)**, GLUT10 **(E)**, GLUT8 **(F)**, GLUT12 **(G)** were measured by real-time quantitative PCR and analyzed by 2^−ΔΔCt^ method (Schmittgen and Livak, 2008). Protein levels of GLUT1 and GLUT12 were determined by immunoblot **(H,I)**. Data are presented as mean ± SEM (*n* = 6 birds/group). Different letters indicate significant difference at *p* < 0.05. GLUT, glucose transporter; NC, negative control; NC + P, negative control supplemented with 2,000 phytase units/kg (FYT per kg of feed); PC, positive control.

As shown in Figure 2A, the tested GLUT (GLUT1, 3, 6, 8, 10, and 12) genes are all expressed in chicken breast muscle. The expression of GLUT1 and GLUT6 was significantly upregulated in phytase-supplemented (NC + P) birds compared to NC and PC groups (Figures 2B, C). Compared to PC birds, GLUT3 mRNA levels were significantly higher in NC, and GLUT10 mRNA abundances were higher in NC + P (Figures 2D, E). mRNA abundances of GLUT8 and GLUT12 were significantly increased in NC compared to PC and NC + P birds (Figures 2F, G). Immunoblot analyses showed that GLUT12 and GLUT1 protein levels were significantly induced in NC + P compared to the other groups (Figures 2H, I).

**FIGURE 3:**
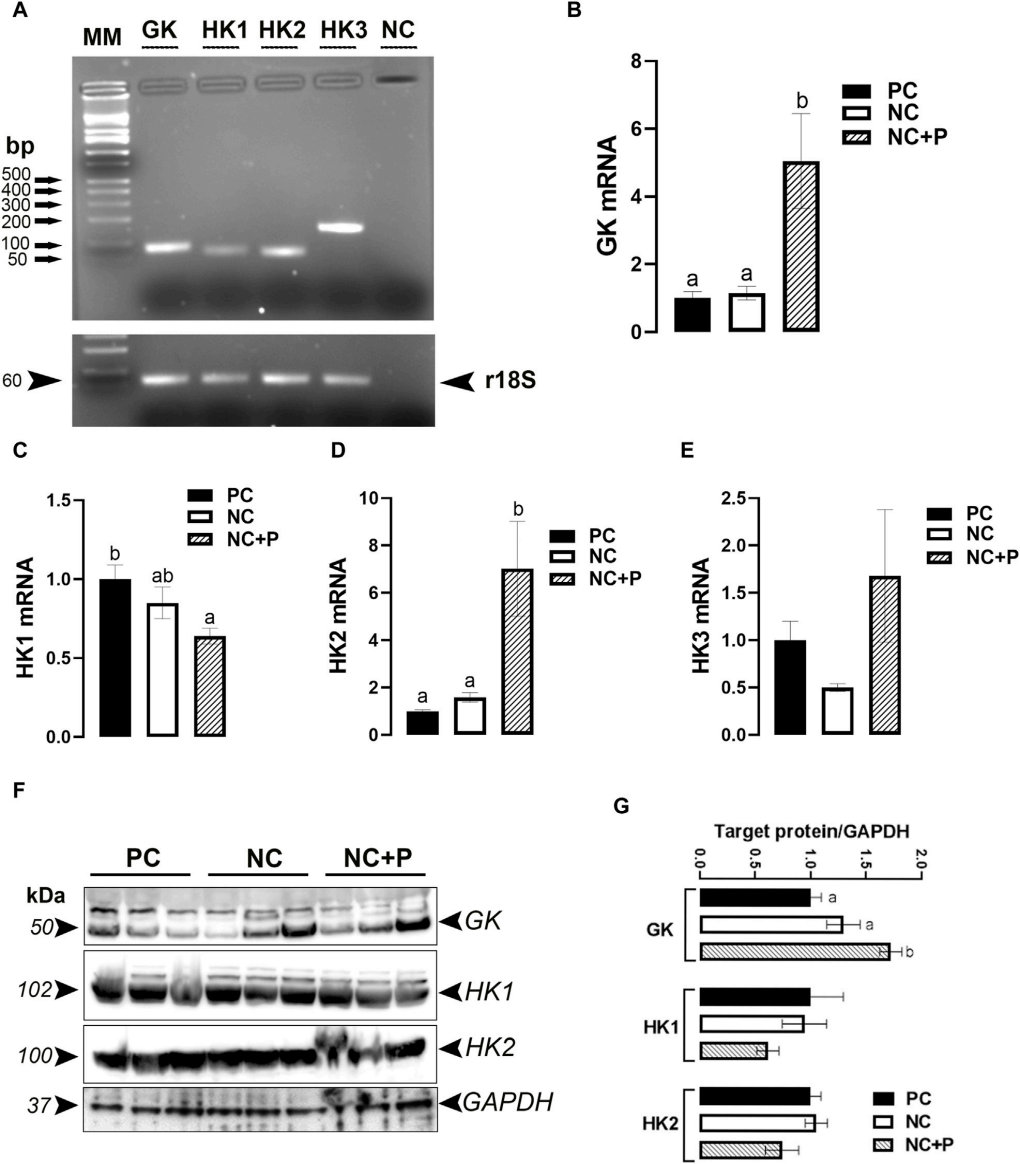
Effect of phytase supplementation on the expression profile of breast muscle hexokinases. Hexokinase-coding genes are expressed in the breast muscle **(A)**. mRNA abundances of GK **(B)**, HK1 **(C)**, HK2 **(D)**, and HK3 **(E)** were measured by real-time qPCR and analyzed by 2^−ΔΔCt^ method (Schmittgen and Livak, 2008). Protein levels of GK, HK1, and HK2 were determined by immunoblot **(F,G)**. Data are presented as mean ± SEM (*n* = 6 birds/group). Different letters indicate significant difference at *p* < 0.05. GK, glucokinase (or HK4), HK, hexokinase; NC, negative control; NC + P, negative control supplemented with 2,000 phytase units/kg (FYT per kg of feed); PC, positive control.

As depicted in Figure 3A, GK (also known as HK4) and HK (1, 2, and 3) genes are all expressed in chicken breast muscle. Phytase supplementation significantly upregulated the expression of GK and HK2 genes, but it downregulated that of HK1 compared to the other groups (Figures 3B–D). The expression of HK3 remained unchanged between all studied groups (Figure 3E). Western blot analyses showed that GK protein levels were significantly increased in NC + P birds compared to the other groups, however HK1 and HK2 remained unchanged between all tested groups (Figures 3F, G).

**FIGURE 4:**
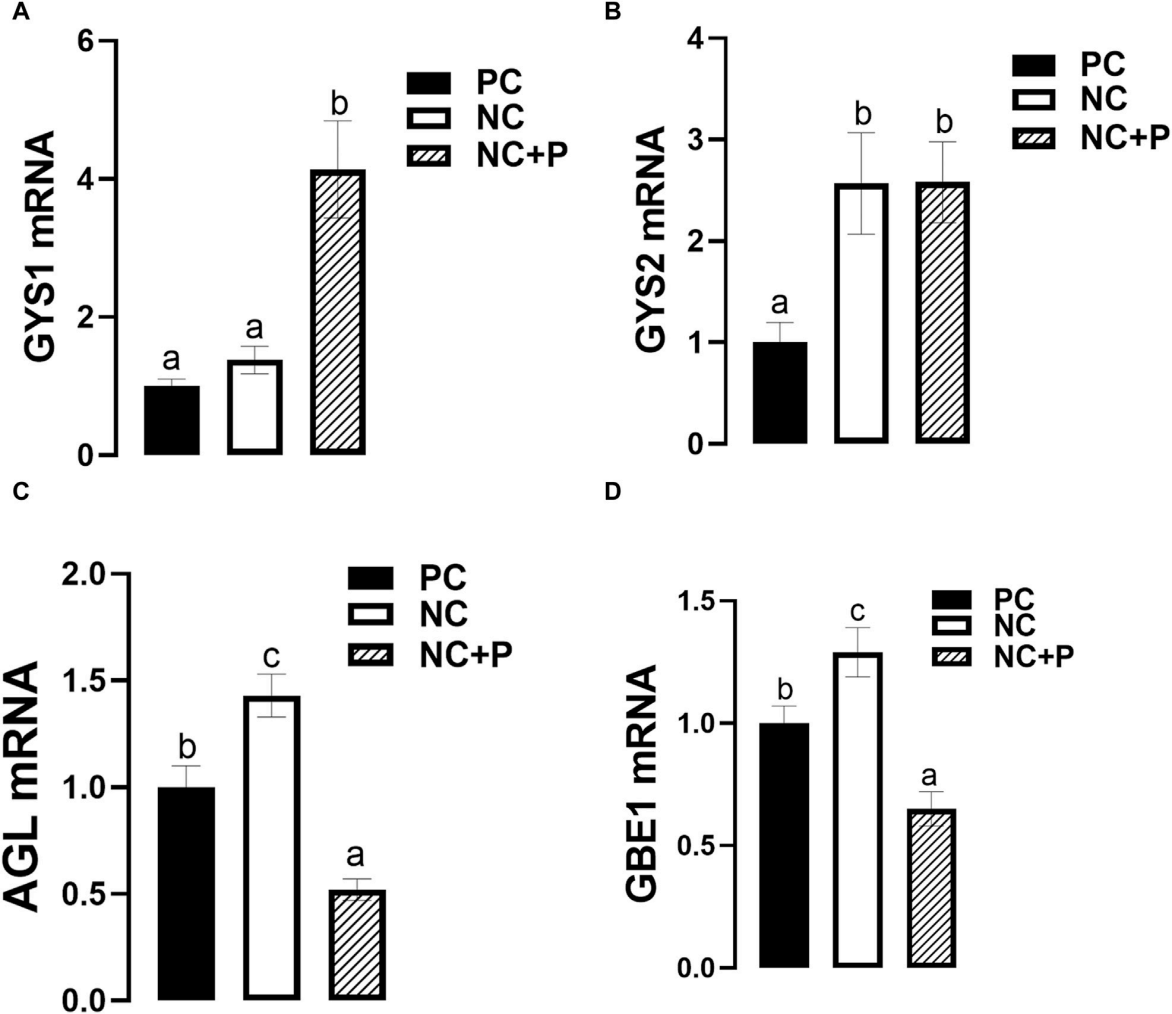
Effect of phytase supplementation on the expression of glycogen metabolism-associated genes in broiler breast muscle. Gene expression of Gys1 **(A)**, Gys2 **(B)**, AGL **(C)**, and GBE1 **(D)** were measured by real-time qPCR and analyzed by 2^−ΔΔCt^ method (Schmittgen and Livak, 2008). Data are presented as mean ± SEM (*n* = 6 birds/group). Different letters indicate significant difference at *p* < 0.05. AGL, glycogen debranching enzyme; GBE1, glycogen branching enzyme; Gys, glycogen synthase; NC, negative control; NC + P, negative control supplemented with 2,000 phytase units/kg (FYT per kg of feed); PC, positive control.

The expression of breast muscle GYS1 was significantly upregulated in NC + P birds compared to the other groups, however GYS2 mRNA levels were significantly higher in both NC and NC + P compared to PC group (Figures 4A, B). Phytase supplementation significantly downregulated the expression of both AGL and GBE1 expression, however NC diet significantly upregulated the expression of the abovementioned genes (Figures 4C, D).

**FIGURE 5:**
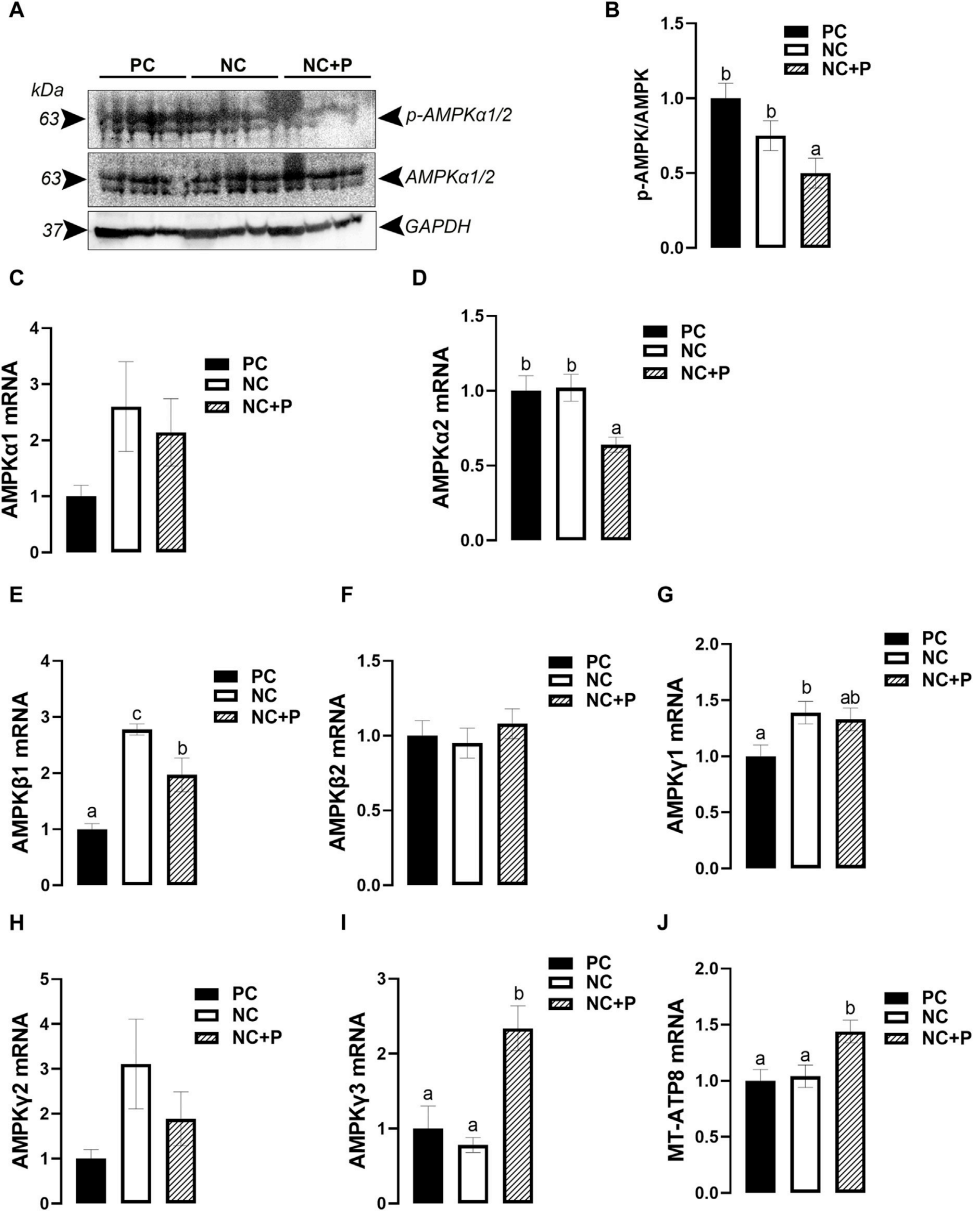
Effect of phytase supplementation on the expression of AMPK subunits in broiler breast muscle. The levels of pan and phosphorylated AMPKα1/2 at Thr172 site were determined by Western blot analysis **(A,B)**. mRNA abundances of AMPKα1 I, AMPKα2 **(D)**, AMPKI **(E)**, AMPKβ2 **(F)**, AMPKγ1 **(G)**, AMPKγ2 **(H)**, AMPKγ3 **(I)**, and MT-ATP8 **(J)** were measured by real-time quantitative PCR and analyzed by 2^−ΔΔCt^ method (Schmittgen and Livak, 2008). Data are presented as mean ± SEM (*n* = 6 birds/group). Different letters indicate significant difference at *p* < 0.05. AMPK, adenosine monophosphate (AMP)-activated protein kinase; MT-ATP8, mitochondrial ATP synthase F0 subunit 8; NC, negative control; NC + P, negative control supplemented with 2,000 phytase units/kg (FYT per kg of feed); PC, positive control.

Phytase supplementation significantly reduced the phosphorylated levels of the catalytic subunit AMPKα1/α2 at Thr172 site, indicating a decreased activity of AMPK in NC + P birds compared to the other groups (Figures 5A, B). Real-time quantitative PCR showed that phytase supplementation significantly downregulated the expression of AMPKα2 and upregulated that of AMPKγ3 and MT-ATP8 compared to PC and NC groups (Figures 5D, I, J). The expression of AMPKβ1was significantly higher in both NC and NC + P compared to PC birds (Figure 5E), however the mRNA abundances of AMPKγ1 was significantly induced only in NC chickens compared to PC group (Figure 5G). The expression of AMPKα1, AMPKβ2, and AMPKγ2 remained unchanged between all studied groups (Figures 5C, F, H).

**FIGURE 6:**
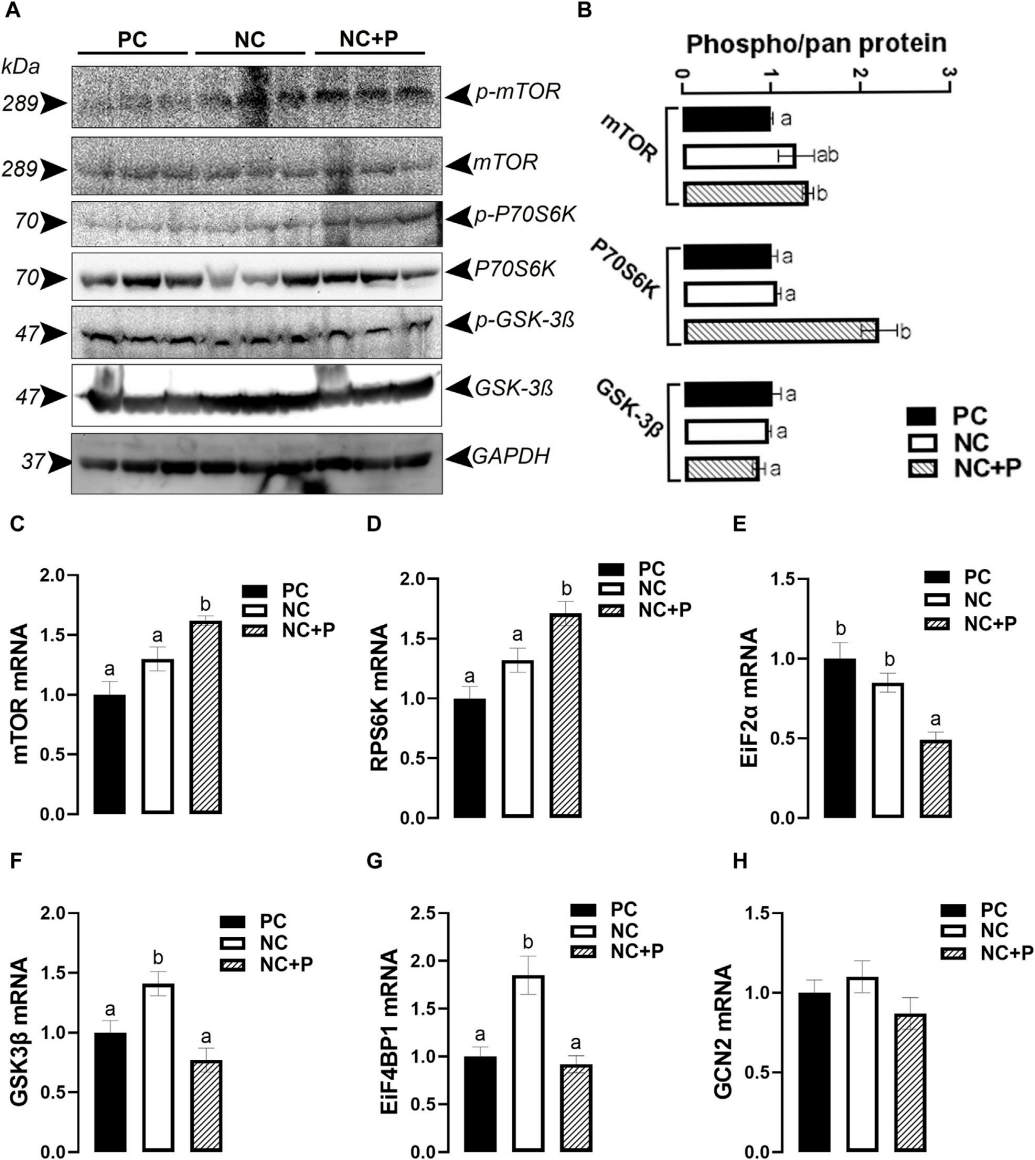
Effect of phytase supplementation on the expression of key genes and proteins involved in breast muscle protein synthesis. The levels of pan and phosphorylated mTOR^Ser2448^, P70S6K^Thr389^, and GSK-3β^Ser9^ were determined by Western blot analysis **(A,B)**. mRNA abundances of mTOR **(C)**, RPS6K **(D)**, EiF2α **(E)**, GSK-3β **(F)**, EiF4BP1 **(G)**, and GCN2 **(H)** were measured by real-time quantitative PCR and analyzed by 2^−ΔΔCt^ method (Schmittgen and Livak, 2008). Data are presented as mean ± SEM (*n* = 6 birds/group). Different letters indicate significant difference at *p* < 0.05. GCN2, general control nonderepressible 2 or eukaryotic translation initiation factor 2 alpha kinase 4; EiF2α, eukaryotic translation initiation factor 2 subunit alpha; EiF4BP1, eukaryotic translation initiation factor 4E binding protein 1; GSK-3β, glycogen synthase kinase 3 beta; mTOR, mechanistic target of rapamycin; P70S6K, ribosomal protein S6 kinase.

Phytase supplementation significantly increased the levels of phosphorylated mTOR and P70S6K at Ser2448 and Thr389 sites, respectively, compared to the other groups (Figures 6A, B). However, the activity of GSK-3β remained unchanged between all tested groups (Figures 6A, B). At mRNA levels, phytase supplementation significantly induced the expression of mTOR and RPS6K, but it reduced that of EiF2α compared to PC and NC diets (Figures 6C–E). The expression of GSK3β and EiF4BP1 was significantly upregulated in NC compared to PC and NC + P birds (Figures 6F, G). GCN2 mRNA levels did not change in all groups (Figure 6H).

## Discussion

The global poultry sector soared from 9 to 133 million tonnes of meat and mounted from 15 to 93 million tonnes of eggs between 1961 and 2020 (Nations and Rome, 2023) and continues to support the livelihoods of billions of people worldwide. However, muscle myopathies, including WB and WS incidence, are emerging on a global scale and imposing heavy welfare and food security burdens on the poultry industry worldwide (Mudalal et al., 2015; Trocino et al., 2015), accounting for more than $1 billion annual economic loss in North America alone (Barbut, 2020). Although their etiologies are still not well defined, breast fillets affected by these myopathies share super-imposed and overlapping histological hallmarks, which are typically characterized by myo-degeneration and necrosis, infiltration of inflammatory cells in the endomysium, and accumulation of connective tissues and fat (Sihvo et al., 2014; Baldi et al., 2018).

Despite their rising incidence and associated complications, mechanistic understanding of these myopathies remains limited. In that regard, several omics studies (Mutryn et al., 2015; Abasht et al., 2016; Kuttappan et al., 2017) and recent functional evidence suggested that hypoxia is a key causative factor for WB (Emami et al., 2021) and that in-feed supplementation of phytase improved growth performance and reduced WB severity (Greene et al., 2019). Building on these previous studies and in a continuum of the same research hot spot, we report here that addition of a new fourth generation phytase improves growth performances (BW, weight corrected FCR, and carcass cut up) and reduces the severity of WB without affecting WS incidence and meat quality attributes (pH and colors) in Ross 708 broilers. This novel phytase was encoded by a 6-phytase gene from *Citrobacter braakii* (ATCC51113), expressed in *Aspergillus oryzae*, and characterized by an optimal enzyme activity and intrinsic temperature- and pH-stability (Imtiaz, 2022; Zhang et al., 2022). The improvement of growth performance observed here is not surprising and it is in agreement with previous investigations (Zhang et al., 2022). However, it is worth noting that compared to the previous study where the effect of dietary phytase on growth was reported to be minimal in older birds (d28-36), here the present study showed that the effect was significant in all rearing phases (ages). Although further studies are needed, the abovementioned differential age-dependent effects were probably associated with the experimental duration (36 days vs. 55 days), conditions and location (China or France vs. United States and cage vs. floor pens), diet composition and nutrient specifications, and/or broiler strains (Cobb 500 and Ross 308 vs. Ross 708) in the previous (Zhang et al., 2022) and in the present experiment, respectively.

In attempt to delineate the mode of action of the novel phytase, and as WB myopathy has been predicted to be associated with carbohydrate dysmetabolism, we next measured blood glucose concentrations using iSTAT Alinity. Interestingly, phytase supplementation decreased circulating glucose levels compared to both NC and PC diets, which suggests an increased glucose uptake by the breast muscle and probably by other glucose-absorbing tissues. The upregulation of GLUT (GLUT1, 6, and 10) gene expression and GLUT1-and GLUT12-protein levels in our experimental conditions supports the abovementioned hypothesis that the novel phytase enhances glucose uptake by the breast muscle (Cowieson et al., 2013; Lu et al., 2019). This has been supported by enriched GLUT1 and GLUT12 proteins in membrane fractions (data not shown). However, it is intriguing and still unknown how and why the other GLUTs (GLUT3, GLUT8, and GLUT12) were induced in the breast muscle of NC birds. As they belong to different classes (I to III) (Olson and Pessin, 1996; Joost and Thorens, 2001), it is possible that these GLUTs have different and/or additional functions and affinities. For instance, GLUT3 has been reported to transport maltose, xylose, dehydroascorbic acid, mannose, and galactose in mammals (Colville et al., 1993). GLUT12, on the other hand, has been shown to be involved in urate transport in rodents (Toyoda et al., 2020). As the glycogen debranching enzyme (AGL) expression was upregulated by the NC diet, it is probable that the activated glycogenolysis pathway might directly or indirectly regulate these GLUTs (Fidler et al., 2017). Furthermore, as we used the whole muscle tissue, it is plausible that the expression of these GLUTs is cell-, compartment-, and/or fiber-specific, and therefore they respond differently to stimuli [for review see (Pyla et al., 2013)].

After transport and uptake, the cellular fate of glucose begins with phosphorylation and then undergoes subsequent utilization pathways, including glycolysis, glycogen formation, or conversion to other intermediates in the hexose phosphate or hexosamine biosynthesis pathways, depending on the expression of isozymic forms of HKs (Bouche et al., 2004). Here, real-time qPCR analyses showed that both GK (also known as HK4) and HK1-3 are expressed in the muscle, corroborating a previous study in chickens (Roy et al., 2013). In human, however, GK expression has been shown to be restricted to the pancreas and the liver (Matschinsky, 1990). Selective expression/regulation of HK isozymic forms, differing in catalytic and regulatory properties as well as subcellular localization, is likely to be an important factor in determining the pattern of glucose metabolism. In fact, despite their overall structural similarity, HK1 (type I) serves for catabolic function for energy production, however HK2 (Type II) and probably HK3 (Type III) isozymes are suggested to serve primarily anabolic functions (Wilson, 2003). The upregulation of GK at both mRNA and protein levels suggests that phytase supplementation induces muscle glycolysis and ATP synthesis, which is supported by the upregulation of MT-ATP8 gene expression and the downregulation of the catalytic AMPKα1/2 subunit gene and its phosphorylated levels at Thr172 site. MT-ATP8 encodes for the mitochondrial ATP synthase (complex V), which is an important enzyme that provides energy to be used by the cell through the ATP synthesis (Jonckheere et al., 2012). AMPK is a conserved master cellular “fuel gauge” and metabolic sensor that is activated by falling energy status, but downregulated when the cellular ATP/AMP ratio is elevated (Hardie et al., 2012; Gowans and Hardie, 2014; Garcia and Shaw, 2017). In most species, AMPK exists as an obligate heterotrimer, containing catalytic subunits (α1 and α2), and two regulatory β (β1 and β2) and γ (γ1, γ2, and γ3) subunits, which are encoded by different genes (Hardie, 2007). The upregulation of both AMPKβ1 and AMPKγ3 subunits by phytase suggests that the downregulation of the catalytic AMPKα1/2 was probably mediated by ATP-binding to these regulatory subunits (Scott et al., 2004). However, it is still unclear why and how the NC diet induces muscle AMPKβ1 and AMPKγ1 expression in our experimental conditions, and the mechanism likely involves calcium flux that remains a critical underexplored area for future investigations (Dunn and Munger, 2020).

The increased level of cellular energy (ATP), described above, can be used for anabolic pathways. As there was no difference in fat content in our experimental conditions, we limited our analyses to glycogen- and protein-biosynthesis. The increased abundance of glycogen synthases (GYS1 and GYS2) mRNA indicates that the supplementation of phytase enhances muscle glycogen synthesis. However, the downregulation of both glycogen branching and debranching enzyme expression suggests that both glycogenolysis and glycogenesis are concomitant, a phenomenon known as glycogen cycling (Landau, 2001). Importantly, it has been shown that AMPKγ3 mutation increased glycogen content and decreased triglyceride in human skeletal muscle (Costford et al., 2007), which suggests that phytase supplementation might regulate breast muscle glycogen and energy homeostasis via AMPKγ3-AMPKα1/2 interaction.

The enhanced BW along with increased part (breast, wing, tender) weights suggest that phytase supplementation enhances global and local (breast muscle) protein synthesis, which is supported by the upregulation of mTOR-RPS6K and the downregulation of EIF-4BP1 and EIF2α expression (Schmeisser et al., 2017). mTOR is a conserved serine/threonine kinase and nutrient sensor (Kim et al., 2002), that belongs to the PI3K-related protein kinase (PIKK) family (Richardson et al., 2004) and plays a key role in ribosomal biogenesis and protein synthesis (Zhang et al., 2014). The well-studied and best-characterized mTOR-downstream cascade is the translation regulator 4E-BP1-S6K1, both of which contain TOR signaling (TOS) motifs (Nojima et al., 2003; Schalm et al., 2003). 4E-BP1 is a translational repressor that inhibits translation initiation by interfering with the interaction between the cap-binding protein eIF4E and eIF4G1 (Hara et al., 1997; Zhang et al., 2010; Thoreen et al., 2012). Activation of EIF2α attenuates global protein synthesis, except the translation of select mRNA such as the activating transcription factor 4 (ATF4) (Harding et al., 2003; Nobukuni et al., 2005). Unlike its inhibitory effect on 4E-BP1 and EIF2α, mTOR activates P70S6K, which in turn, regulates ribosome function, mRNA processing, cap-dependent translation initiation and elongation, and then promotes several cellular processes, including protein production and cell size/growth (Price et al., 1992; Isotani et al., 1999; Magnuson et al., 2012). Although the exact mechanism by which phytase supplementation activates mTOR is not known at this time point, it is possible that the effect is mediated through high amino acid digestibility, absorption, and utilization (Nobukuni et al., 2005; Zoncu et al., 2011; Manifava et al., 2016). Indeed, exogenous phytase fed at high dose has been reported to induce a rapid and complete breakdown of phytate in diets, reducing the anti-nutritional effects, and thereby eliciting a greater amino acid utilization (Amerah et al., 2014; Sommerfeld et al., 2018; Siegert et al., 2021). Moreover, it is plausible that phytase activates mTOR via available phosphorus-stimulating AKT pathway (Simons et al., 1990; Jin et al., 2009; Liu et al., 2017). Although it was not functionally demonstrated here, it is very likely that phytase activates mTOR via glucose-AMPK pathway (Leprivier and Rotblat, 2020; Rumala et al., 2020; Caligaris et al., 2023), which merits future in-depth investigation. It is logical also to hypothesize that phytase supplementation might activate mTOR pathway via increased inositol (Kim et al., 2011; Badodi et al., 2021) and/or improvement of oxygen homeostasis (Miloslavski et al., 2014; Greene et al., 2019; Zhu et al., 2020), that warrants further consideration.

In summary, this is the first report, to the best of our knowledge, showing potential new mechanisms evolving glucose metabolism and AMPK-mTOR pathway, by which the novel phytase improves growth and reduces WB myopathy. This opens a new vista and provides a framework for future in-depth mechanistic research using different chicken strains.

## Data Availability

The original contributions presented in the study are included in the article/Supplementary Material, further inquiries can be directed to the corresponding author.
