# Supplementary material for: Novel 4th-generation phytase improves broiler growth performance and reduces woody breast severity through modulation of muscle glucose uptake and metabolism
Source: Front Physiol. 2024 Mar 15;15:1376628. doi: 10.3389/fphys.2024.1376628 (PMC10978611; doi:10.3389/fphys.2024.1376628)
Supplement: Supplementary file 1 [file DataSheet1.PDF]

Novel 4th-generation phytase improves broiler growth performance through modulation of muscle glucose uptake and metabolism

Carrie, L. Walk[1], Garrett, J. Mullenix[2,#], Craig, W. Maynard[2,$], Elisabeth, S. Greene[2], Clay Maynrad[2], Nelson Ward[3], Sami Dridi[2*]

[1]DSM Nutritional Products, 4303 Kaiseraugst, Switzerland

[2]Department of Poultry Science, University of Arkansas, Fayetteville, AR 72701, USA

[3]DSM Nutritional Products, Jerusalem, OH, 43747, USA

[#]Actual address: Cargill Inc., Plymouth, MN 55441, USA

[$]Actual address: Bell and Evans, Fredericksburg, PA 17026, USA

*Corresponding author: Sami Dridi, Center of Excellence for Poultry Science, University of Arkansas, 1260 W. Maple Street, Fayetteville, AR 72701, USA

Phone: (479)-575-2583, Fax: (479)-575-7139, email: dridi@uark.edu

Figure S1

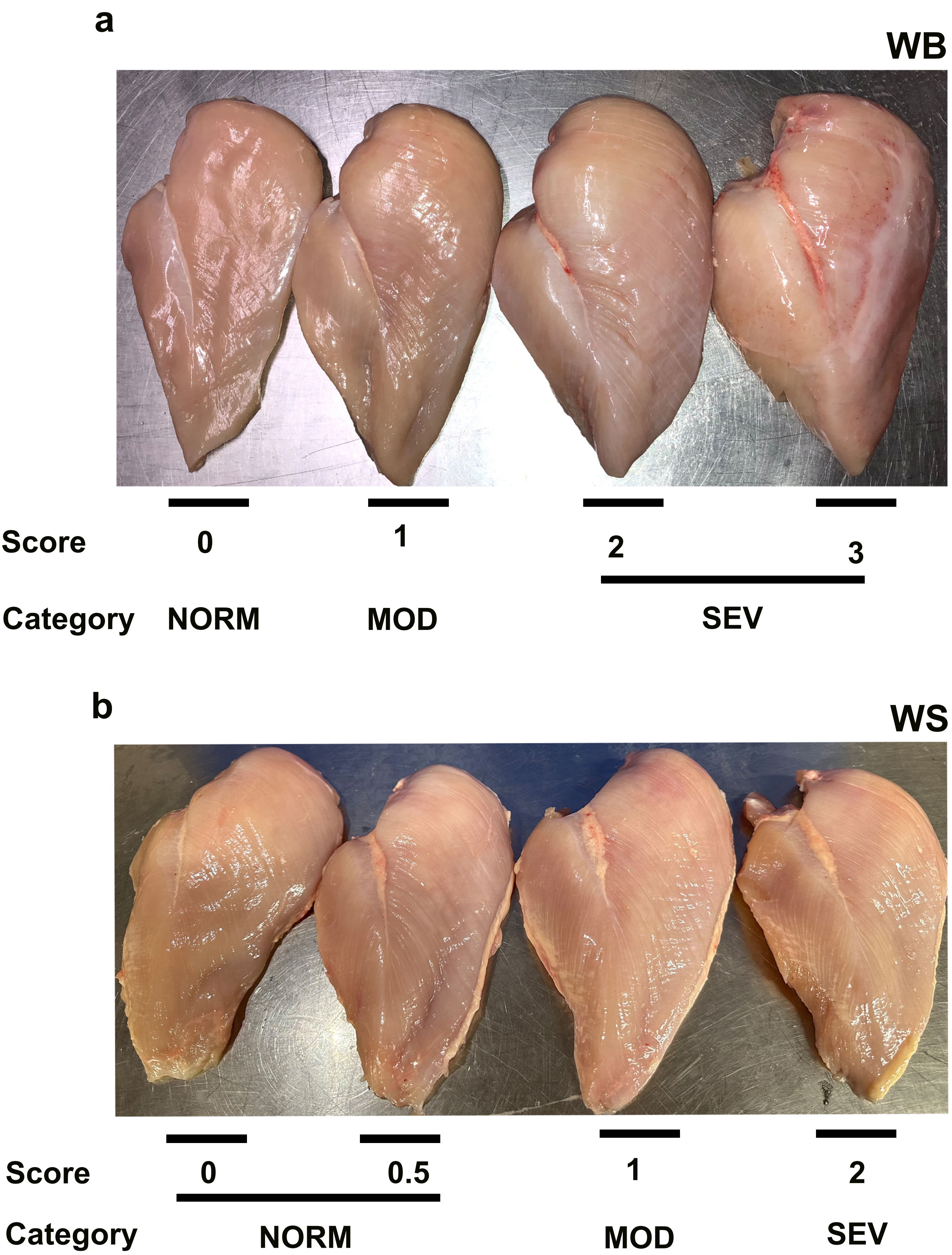
a
WB
Score 0 1 2 3
Category NORM MOD SEV
b
WS
Score 0 0.5 1 2
Category NORM MOD SEV
